# Variability over time and correlates of cholesterol and blood pressure in systemic lupus erythematosus: a longitudinal cohort study

**DOI:** 10.1186/ar3063

**Published:** 2010-06-30

**Authors:** Mandana Nikpour, Dafna D Gladman, Dominique Ibanez, Paula J Harvey, Murray B Urowitz

**Affiliations:** 1 University of Toronto Lupus Clinic and the Centre for Prognosis Studies in the Rheumatic Diseases, Toronto Western Hospital, 399 Bathurst Street, Toronto, ON, M5T 2S8, Canada; 2 University of Melbourne Department of Medicine, St. Vincent's Hospital, 41 Victoria Parade, Fitzroy, Melbourne, Victoria, 3065, Australia; 3 Division of Cardiology and Clinical Pharmacology, Toronto Western Hospital, 399 Bathurst Street, Toronto, ON, M5T 2S8, Canada

## Abstract

**Introduction:**

Total cholesterol (TC) and blood pressure (BP) are likely to take a dynamic course over time in patients with systemic lupus erythematosus (SLE). This would have important implications in terms of using single-point-in-time measurements of these variables to assess coronary artery disease (CAD) risk. The objective of this study was to describe and quantify variability over time of TC and BP among patients with SLE and to determine their correlates.

**Methods:**

Patients in the Toronto lupus cohort who had two or more serial measurements of TC and systolic and diastolic BP (SBP and DBP) were included in the analysis. Variability over time was described in terms of the proportion of patients whose TC and BP profile fluctuated between normal and elevated (TC > 5.2 mmol/L; SBP ≥ 140 mm Hg *or *DBP ≥ 90 mm Hg), and also in terms of within- and between-patient variance quantified by using analysis of variance modeling. Generalized estimating equations (GEEs) were used to determine independent correlates of each of TC, SBP, and DBP, treated as continuous outcome variables.

**Results:**

In total, 1,260 patients, comprising 26,267 measurements of each of TC, SBP, and DBP, were included. Mean ± SD number of measurements per patient was 20.8 ± 20. Mean ± SD time interval between measurements was 5.4 ± 9.7 months. Mean ± SD time interval from the start to the end of the study was 9.3 ± 8.5 years. Over time, 64.7% of patients varied between having normal and elevated cholesterol levels, whereas the status of 46.4% of patients varied between normotensive and hypertensive. By using analysis of variance (ANOVA), the *within-patient *percentage of total variance for each of TC, SBP, and DBP was 48.2%, 51.2%, and 63.9%, respectively. By using GEE, independent correlates of TC and BP included age, disease activity, and corticosteroids; antimalarial use was negatively correlated with TC (all *P *values < 0.0001).

**Conclusions:**

TC and BP vary markedly over time in patients with SLE. This variability is due not only to lipid-lowering and antihypertensive medications, but also to disease- and treatment-related factors such as disease activity, corticosteroids, and antimalarials. The dynamic nature of TC and BP in SLE makes a compelling case for deriving summary measures that better capture cumulative exposure to these risk factors.

## Introduction

Systemic lupus erythematosus (SLE) is strongly associated with premature atherosclerotic CAD [1,2]. Indeed, young women aged 35 to 44 years are > 50 times more likely to have myocardial infarction than are their age-matched peers [3]. One in 10 patients with SLE is diagnosed with clinical CAD, making this complication one of the leading causes of morbidity and mortality in SLE [4,5]. Whilst traditional cardiovascular risk factors only partly account for the increased risk of CAD in SLE, many of these risk factors are potentially treatable [6].

Hypercholesterolemia and hypertension are two traditional cardiac risk factors that have been shown to be independently predictive of coronary events in patients with SLE when measured at the first available visit ('baseline') or defined as 'abnormal ever' during follow-up [3,4,7]. However, to date, the magnitude of risk associated with these risk factors may not have been accurately estimated by using approaches that fail to take into account the possible variability of these risk factors over time.

Evidence suggests that in the first 3 years of disease, one third of patients with SLE have 'variable hypercholesterolemia', with cholesterol levels that fluctuate between 'normal' and 'abnormal', which, in this case, is defined as total serum cholesterol > 5.2 mmol/L [8]. Similarly, in the general population, systolic and diastolic blood pressure have been shown to vary over time, a phenomenon that likely also affects SLE patients in whom both disease manifestations and treatments may affect blood pressure [9-11]. To date, the variability over time of TC, SBP, and DBP over the course of disease in patients with SLE has not been rigorously evaluated. The objective of this study was to describe and quantify variability over time of TC, SBP, and DBP and to determine their correlates in patients with SLE. We used > 26,000 measurements of each of TC, SBP, and DBP taken in > 1,200 SLE patients, in > 9 years of follow-up. In assessment of variability over time, we defined each of TC, SBP, and DBP dichotomously *and *as continuous variables. Generalized estimating equations (GEEs) were used to determine independent correlates of TC, SBP, and DBP over time.

## Materials and methods

### Patients

Among the University of Toronto lupus cohort, patients who had two or more *serial *measurements of TC, SBP, and DBP were included in the analysis. Patients attending the University of Toronto lupus clinic are followed up at 2- to 6-month intervals, and clinical and laboratory data obtained at each visit are stored in a dedicated database. All patients fulfill four or more of the ACR classification criteria for SLE, or have three criteria and a typical lesion of SLE on renal or skin biopsy [12,13]. Collection and storage of data are approved by the research ethics board of the University Health Network, and patients give informed consent on entry into the clinic.

### Methods

#### TC, SBP, and DBP and 'other' variables

In addition to TC, SBP, and DBP, data on patients' demographic profiles (including age, sex, menopausal status, and race), disease duration, disease activity, medications, intercurrent infections, smoking, and diabetes were routinely collected according to a set protocol. The data were stored and tracked in the lupus database at each clinic visit for the period from entry into the clinic up to the most recent visit as of August 2008. Each measurement of TC, SBP, and DBP was therefore tied to a clinic visit. We used only visits wherein all of three of TC, SBP, and DBP had been measured and recorded.

##### Definitions of variables

Age and disease duration at the time of each visit were reported in years. Disease duration was calculated from the date of physician diagnosis of SLE to the date of each visit. Disease activity at each visit was reported by using the SLE Disease Activity Index 2000 (SLEDAI-2K), wherein scores range from 0 to 105, with higher scores indicating more-active disease [14]. Corticosteroid, antimalarial, and immunosuppressive use at each visit were reported categorically, irrespective of dose. Antimalarials included chloroquine and hydroxychloroquine. Immunosuppressives included methotrexate, azathioprine, mycophenolate mofetil, cyclosporine, and cyclophosphamide. Antihypertensives included all classes of drugs used to reduce blood pressure. Lipid-lowering medications were predominantly 'statins.' Antihypertensive and lipid-lowering therapy at each visit was defined categorically. TC level was measured nonfasting in plasma by using a commercial assay (kit 236691; Boehringer Mannheim, Indianapolis, IN) at each visit and recorded in millimoles per liter (mmol/L). It has been shown that only small, clinically insignificant differences in cholesterol level are found when measured in the fasting or nonfasting state [15].

Hypercholesterolemia was defined as total plasma cholesterol > 5.2 mmol/L [8,16]. SBP and DBP were measured in millimeters of mercury (mm Hg) at each visit by using a manual sphygmomanometer. Hypertension was defined as DBP ≥ 90 or SBP ≥ 140 mm Hg [17]. Diabetes was defined as fasting plasma glucose > 7.0 mmol/L or diabetes therapy. Menopause was defined as a minimum of 12 months of amenorrhea, irrespective of cause. Hormone-replacement therapy was defined as treatment with estrogen with or without progestin.

### Statistical analysis

Characteristics of patients in the study as well as the total number, frequency, and values of TC, SBP, and DBP measurements are described. The proportion of patients with 'normal' or 'elevated' TC, SBP, and DBP at study entry and during follow-up was determined. 'Method of moments' analysis of variance (ANOVA) modeling was used to quantify total, within-, and between-patient variance in TC, SBP, and DBP, each treated as a *continuous *variable.

Linear regression modeling with analysis of repeated measures was performed by using GEE to determine the independent correlates of each of TC, SBP, and DBP ('outcome' variables). Predictor/independent variables ('covariates') included sex, age, disease duration, SLEDAI-2K score, infection, diabetes, smoking, and treatment with corticosteroids, antimalarials, immunosuppressives, antihypertensives, and lipid-lowering medications. For each covariate, the measurements used were those recorded at the time of (that is, 'coincident') with each measurement of SBP or DBP.

In the model used to determine correlates of TC, hypertension was also included as a covariate, whereas in the models used to determine correlates of SBP and DBP, hypercholesterolemia was also included as a covariate. Modeling was repeated by using only female patients. In these models, in addition to the aforementioned independent variables, menopausal status and hormone-replacement therapy were also included as covariates.

All statistical analyses were performed by using SAS version 9.1 (SAS Institute Inc., Cary, NC).

## Results

In total, 1,260 patients were included in the analysis, comprising 26,267 measurements of each of TC, SBP, and DBP. The characteristics of these patients are summarized in Table 1. The patients were mostly female (88.3%) and white (73%). Among the female patients, 224 (20.1%) were menopausal at study entry, and 445 (40.0%) were menopausal either at study entry or during follow-up. Mean ± standard deviation (SD) age at first clinic visit and at entry to study were 35.0 ± 13.6 and 35.4 ± 13.7 years, respectively. In 80% of patients, the first clinic visit was also the entry visit into the study. Mean ± SD disease duration at first clinic visit and at entry to study were 4.0 ± 5.0 and 4.4 ± 6.0 years, respectively. Among the patients, 42% had their first study visit within 12 months of diagnosis ('inception cohort'). Among noninception patients, at the first study visit, mean ± SD disease duration was 7.3 ± 6.4 years, ranging from 1 to 52 years. Mean ± SD SLEDAI-2K score at first clinic visit and at entry to study were 9.6 ± 7.7 and 8.7 ± 7.0, respectively, indicating moderate disease activity.

**Table 1:** Characteristics of patients (*n *= 1,260)

Characteristic	Number (%) or mean ± SD
Female	1,113 (88.3%)
Menopausal at entry to study^a^	224 (20.1%)
Menopausal during follow-up^a^	445 (40.0%)
Race: White	880 (73%)
Black	119 (10%)
Asian	113 (9%)
Other	96 (8%)
Age at first clinic visit (years)	35.0 ± 13.6
Disease duration at first clinic visit (years)	4.0 ± 5.0
SLEDAI-2K at first clinic visit^b^	9.6 ± 7.7
Age at entry to study (years)	35.4 ± 13.7
Disease duration at entry to study (years)	4.4 ± 6.0
SLEDAI-2K at entry to study^b^	8.7 ± 7.0
Hypertension at entry to study^c^	190 (15.1%)
Hypercholesterolemia at entry to study^e^	528 (41.9%)
Diabetes at entry to study^f^	30 of 1,223 (2.5%)^d^
Smoker at entry to study^g^	247 of 1,235 (20.0%)^d^
Corticosteroid use at entry to study	763 of 1,257 (60.7%)^d^
Antimalarial use at entry to study^h^	462 of 1,256 (36.8%)^d^
Immunosuppressive use at entry to study^i^	259 of 1,255 (20.6%)^d^

SD, standard deviation.

^a^Menopause defined as a minimum of 12 months of amenorrhea, irrespective of cause.

^b^Scores range from 0 to 105, with higher scores indicating more-active disease.

^c^Diastolic BP ≥ 90 or systolic BP ≥ 140 mm Hg.

^d^For these variables, data were incomplete for a small number of patients. The denominator of the fractions in the second column is the total number of patients from whom the percentage was calculated.

^e^Hypercholesterolemia was defined as cholesterol > 5.2 mmol/L.

^f^Diabetes was defined as fasting plasma glucose > 7.0 mmol/L or diabetes therapy.

^g^Smoking one or more cigarettes per day.

^h^Antimalarials include chloroquine and hydroxychloroquine.

^i^Immunosuppressives include methotrexate, azathioprine, mycophenolate mofetil,

cyclosporine, and cyclophosphamide.

The total number, frequency, and values of TC, SBP, and DBP measurements are reported in Table 2. For each of TC, SBP, and DBP, the mean ± SD and median number of measurements per patient were 20.8 ± 20.8 and 14, respectively. The mean ± SD and median time interval between measurements were 5.6 ± 9.7 and 3.7 months, respectively. The mean ± SD and median time interval from the start to the end of the study were 9.3 ± 8.5 and 6.5 years, respectively. The mean ± SD level of TC at the start of study was 5.2 ± 1.7 mmol/L. The mean ± SD level of SBP at the start of the study was 123 ± 19.2 mm Hg. The mean ± SD level of DBP at the start of study was 77.2 ± 12.0 mm Hg.

**Table 2:** Number, frequency, and values of total cholesterol (TC), systolic blood pressure (SBP), and diastolic blood pressure (DBP) measurements

	Mean ± SD	Min, Max	Median
Number of measurements per patient	20.8 ± 20.8	2, 124	14
Time interval between visits (months)	5.6 ± 9.7	0.13, 338.3	3.7
Time from study start to end (years)	9.3 ± 8.5	0.1, 35.0	6.5
TC at start of study (mmol/L)	5.2 ± 1.7	1.1, 16.1	4.9
SBP at start of study (mm Hg)	123 ± 19.2	80, 220	120
DBP at start of study (mm Hg)	77.2 ± 12.0	55, 180	80

SD, standard deviation; Min, Max, minimum and maximum.

The proportion of patients with normal (or elevated) TC or BP at the start of the study and during follow-up is reported in Table 3. Of note, over time, 64.7% of patients varied between having normal and elevated TC levels, with hypercholesterolemia recorded for 36% of the total number of visits. Likewise, the status of 46.4% of patients varied between normotensive and hypertensive, with hypertension recorded for 14% of the total number of visits.

**Table 3:** Proportion of patients with normal and elevated^a ^total cholesterol (TC), systolic blood pressure (SBP), and diastolic blood pressure (DBP) at baseline and during follow-up

Variable	Elevated at study start *n *(%)	Persistently normal *n *(%)	Persistently elevated *n *(%)	Varying *n *(%)	Visits elevated (%)
TC^a^	528 (41.9)	334 (26.5)	111 (8.8)	815 (64.7)	36
SBP (mm Hg)	153 (12.1)	725 (58.0)	15 (1.2)	520 (41.3)	12
DBP (mm Hg)	114 (9.1)	804 (64.0)	7 (0.6)	449 (35.6)	7
BP (mm Hg)	190 (15.1)	654 (51.9)	21 (1.7)	585 (46.4)	14

^a^Elevated TC is defined as > 5.2 mmol/L. Elevated SBP is defined as ≥ 140 mm Hg. Elevated DBP is defined as ≥ 90 mm Hg. Elevated BP is defined as either SBP ≥ 140 mm Hg or DBP ≥ 90 mm Hg.

The total and the within- and between-patient variance in TC, SBP, and DBP determined by using method of moments ANOVA is reported in Table 4. In this analysis, the TC, the SBP, and the DBP were treated as continuous variables. In the case of TC, 51.8% of the total variance was attributable to variance between patients, whereas 48.2% of the total variance was seen within individuals. For SBP, 48.8% of the total variance was due to variance between patients, whereas 51.2% of the total variance was seen within patients. Similarly for DBP, between-patient variance comprised 36.1% of the total variance, whereas with-in patient variance accounted for 63.9% of the total variance.

**Table 4:** Total, between-, and within-patient variance in total cholesterol (TC), systolic blood pressure (SBP), and diastolic blood pressure (DBP) during follow-up

	Total variance	Between-patient variance	Within-patient variance	Variance between patients (%)	Variance within patients (%)
TC (mmol/L)	1.9	0.97	0.91	51.8	48.2
SBP (mm Hg)	347.3	169.6	177.7	48.8	51.2
DBP (mm Hg)	119.2	43.1	76.1	36.1	63.9

Linear-regression modeling with repeated measures analysis using GEE revealed several independent correlates of TC (Table 5): coincident age (parameter estimate, 0.009; 95% confidence interval (CI) 0.004 to 0.014; *P *= 0.0005), coincident SLEDAI-2K score (parameter estimate, 0.04; 95% CI, 0.03 to 0.05; *P *< 0.0001); coincident corticosteroid use (parameter estimate, 0.32; 95% CI, 0.22 to 0.42; *P *< 0.0001); coincident use of immunosuppressives (parameter estimate, 0.17; 95% CI, 0.06 to 0.27; *P *= 0.0017); coincident use of antihypertensives (parameter estimate, 0.19; 95% CI, 0.08 to 0.30; *P *= 0.0009); and coincident hypertension (parameter estimate, 0.34; 95% CI, 0.22 to 0.46; *P *< 0.0001). Coincident use of antimalarials was negatively correlated with TC (parameter estimate, -0.42; 95% CI, -0.53 to -0.32; *P *< 0.0001). When the model was run with only female patients (Table 6), in addition to the variables listed, another independent correlate of TC was coincident hormone-replacement therapy (parameter estimate, 0.17; 95% CI, 0.09 to 0.25; *P *< 0.0001). A trend toward a significant association with menopausal status was noted (*P *= 0.089). Disease duration (parameter estimate, -0.004; 95% CI, -0.006 to -0.0017; *P *= 0.0008) and coincident lipid-lowering therapy (parameter estimate, -0.09; 95% CI, -0.15 to -0.03; *P *= 0.004) were negatively correlated with TC.

**Table 5:** Independent correlates of total cholesterol determined by using multivariate linear regression (GEE)

Variable^a^	Parameter estimate	95% CI	*P *value
Age (years)	0.009	0.004, 0.014	0.0005
SLEDAI-2K score^b^	0.04	0.03, 0.05	< 0.0001
Corticosteroids	0.32	0.22, 0.42	< 0.0001
Antimalarials^c^	-0.42	-0.53, -0.32	< 0.0001
Immunosuppressives^d^	0.17	0.06, 0.27	0.0017
Antihypertensives^e^	0.19	0.08, 0.30	0.0009
Hypertension^f^	0.34	0.22, 0.46	< 0.0001

GEE, generalized estimating equation; CI, confidence interval.

^a^All variables measured coincident with measurement of total cholesterol.

^b^SLE Disease Activity Index 2000; scores range from 0 to 105, with higher scores indicating more-active disease.

^c^Antimalarials include chloroquine and hydroxychloroquine.

^d ^Immunosuppressives include methotrexate, azathioprine, mycophenolate mofetil,

cyclosporine, and cyclophosphamide.

^e^Antihypertensives include all classes of drugs used to lower blood pressure.

^f^Hypertension is defined as systolic BP ≥ 140 mm Hg or diastolic BP ≥ 90 mm Hg.

**Table 6:** Independent correlates of total cholesterol in women only, determined by using multivariate linear regression (GEE)

Variable^a^	Parameter estimate	95% CI	*P *value
Age (years)	0.009	0.006, 0.011	< 0.0001
SLEDAI-2K score^b^	0.04	0.036, 0.046	< 0.0001
Disease duration (years)	-0.004	-0.006, -0.0017	0.0008
Corticosteroids	0.31	0.26, 0.36	< 0.0001
Antimalarials^c^	-0.41	-0.45, -0.36	< 0.0001
Immunosuppressives^d^	0.15	0.11, 0.20	< 0.0001
Antihypertensives^e^	0.19	0.14, 0.24	< 0.0001
Hypertension^f^	0.25	0.19, 0.32	< 0.0001
Lipid-lowering meds (statins)	-0.09	-0.15, -0.03	0.004
HRT^g^	0.17	0.09, 0.25	< 0.0001

GEE, generalized estimating equation; CI, confidence interval.

^a^All variables measured coincident with measurement of total cholesterol.

^b^SLE Disease Activity Index 2000; scores range from 0 to 105, with higher scores indicating more-active disease.

^c^Antimalarials include chloroquine and hydroxychloroquine.

^d ^Immunosuppressives include methotrexate, azathioprine, mycophenolate mofetil,

cyclosporine, and cyclophosphamide.

^e^Antihypertensives include all classes of drugs used to lower blood pressure.

^f^Hypertension is defined as systolic BP ≥ 140 mm Hg or diastolic BP ≥ 90 mm Hg.

^g^Estrogen with/without progestin hormone-replacement therapy.

Independent correlates of SBP determined by using GEE are listed in Table 7. Overall SBP was independently correlated with coincident age (parameter estimate, 0.41; 95% CI, 0.35 to 0.48; *P *< 0.0001), SLEDAI-2K score (parameter estimate, 0.39; 95% CI, 0.28 to 0.50; *P *< 0.0001), use of antihypertensives (parameter estimate, 6.44; 95% CI, 4.94 to 7.94; *P *< 0.0001), and hypercholesterolemia (parameter estimate, 3.78; 95% CI, 2.50 to 5.05; *P *< 0.0001). When the model was run using only female patients (Table 8), in addition to these variables, other independent correlates of SBP were diabetes (parameter estimate, 2.43; 95% CI, 1.16 to 3.70; *P *= 0.0002) and coincident smoking (parameter estimate, 1.12; 95% CI, 0.20 to 2.04; *P *= 0.017). A trend was noted toward a significant association with menopausal status (*P *= 0.0927). Coincident use of antimalarials (parameter estimate, -1.32; 95% CI, -1.96 to -0.69; *P *< 0.0001), immunosuppressives (parameter estimate, -1.81; 95% CI, -2.48 to -1.13; *P *< 0.0001) and lipid-lowering therapy (parameter estimate, -1.62; 95% CI, -2.52 to -0.73; *P *= 0.0004) were negatively correlated with SBP.

**Table 7:** Independent correlates of systolic blood pressure determined by using multivariate linear regression (GEE)

Variable	Parameter estimate	95% CI	*P *value
Age (years)	0.41	0.35, 0.48	< 0.0001
SLEDAI-2K score^a^	0.39	0.28, 0.50	< 0.0001
Antihypertensives	6.44	4.94, 7.94	< 0.0001
Hypercholesterolemia	3.78	2.50, 5.05	< 0.0001

GEE, generalized estimating equations; CI, confidence interval.

All variables were measured coincident with measurement of total cholesterol.

^a^SLE Disease Activity Index 2000; scores range from 0 to 105, with higher scores indicating more-active disease.

Antihypertensives include all classes of drugs used to lower blood pressure.

Hypercholesterolemia defined as total plasma cholesterol > 5.2 mmol/L.

**Table 8:** Independent correlates of systolic blood pressure in women only, determined by using multivariate linear regression (GEE)

Variable	Parameter estimate	95% CI	*P *value
Age (years)	0.44	0.40, 0.48	< 0.0001
SLEDAI-2K score^a^	0.37	0.30, 0.44	< 0.0001
Antimalarials^b^	-1.32	-1.96, -0.69	< 0.0001
Immunosuppressives^c^	-1.81	-2.48, -1.13	< 0.0001
Antihypertensives^d^	6.85	6.17, 7.53	< 0.0001
Diabetes^e ^	2.43	1.16, 3.70	0.0002
Smoking^f^	1.12	0.20, 2.04	0.017
Hypercholesterolemia^g^	3.10	2.41, 3.78	< 0.0001
Lipid-lowering meds (statins)	-1.62	-2.52, -0.73	0.0004

GEE, generalized estimating equations; CI, confidence interval.

All variables were measured coincident with measurement of total cholesterol.

^a^SLE Disease Activity Index 2000; scores range from 0 to 105, with higher scores indicating more-active disease.

^b^Antimalarials include chloroquine and hydroxychloroquine.

^c^Immunosuppressives include methotrexate, azathioprine, mycophenolate mofetil,

cyclosporine, and cyclophosphamide.

^d^Antihypertensives include all classes of drugs used to lower blood pressure.

^e^Diabetes is defined as fasting plasma glucose > 7.0 mmol/L or diabetes therapy.

^f^Smoking one or more cigarettes per day.

^g^Hypercholesterolemia defined as total plasma cholesterol > 5.2 mmol/L.

Independent correlates of DBP determined by using GEE overall mirrored those of SBP. DBP was independently correlated with coincident age (parameter estimate, 0.08; 95% CI, 0.04 to 0.11; *P *= 0.0001), SLEDAI-2K score (parameter estimate, 0.23; 95% CI, 0.16 to 0.30; *P *< 0.0001), coincident use of antihypertensives (parameter estimate, 3.75; 95% CI, 2.83 to 4.66; *P *< 0.0001) and coincident hypercholesterolemia (parameter estimate, 2.60; 95% CI, 1.83 to 3.38; *P *< 0.0001). When the model was run using only female patients, in addition to these variables, coincident disease duration (parameter estimate, 0.03; 95% CI, 0.01 to 0.05; *P *= 0.008) also was independently correlated with DBP. Coincident use of antimalarials (parameter estimate, -0.94; 95% CI, -1.36 to -0.52; *P *< 0.0001), immunosuppressives (parameter estimates, -0.50; 95% CI, -0.94 to -0.05; *P *= 0.028), and lipid-lowering therapy (parameter estimate, -1.13; 95% CI, -1.72 to -0.53; *P *= 0.0002) were negatively correlated with DBP.

## Discussion

This study revealed substantial changes in TC, SBP, and DBP level over time among patients with SLE. Multivariate regression analysis using GEE showed an association of TC, SBP, and DBP, not only with lipid-lowering and antihypertensive therapy, but also with lupus activity and medications and other cardiovascular risk factors.

This study of variability and correlates of TC and BP was based on numerous (on average, 20) and frequent (on average, every 5.6 months) measurements of these variables in 1,260 patients with SLE, followed up on average for 9.3 years. In total, a large dataset of 26,267 individual data points was used in analysis of variability and correlates for TC, SBP, and DBP.

We chose to report 'variability' in serial measurements taken over time in two ways. First, TC, SBP, and DBP each were dichotomized into 'normal' and 'elevated' values based on conventional cut points, and over time, the proportion of patients in whom values fluctuated from one category to another was determined. Second, with TC, SBP, and DBP treated as continuous variables, total variance in each variable was quantified and dissected into within- and between-patient variance by using ANOVA modeling. The latter approach eliminates the need to dichotomize TC and BP values according to cut points, which, although based on evidence, are somewhat arbitrary. Common to both methods is the assessment of change in mean or average values over time. However, it must be borne in mind that this approach does not capture the trajectory taken by each variable measured serially in each patient.

In this study, over a mean and median follow-up period of 9.3 and 6.5 years, respectively, 8.8% of patients had persistent hypercholesterolemia, whereas almost two thirds (64.7%) had variable hypercholesterolemia. This is even greater variability over time than previously reported in SLE patients in the first 3 years of disease, wherein one third of patients had persistent hypercholesterolemia, whereas one third had variable hypercholesterolemia [8]. The greater variability and fewer cases of persistent elevation in cholesterol may be due to fluctuations in disease activity over time and the effect of changes to therapy, including the use of corticosteroids and lipid-lowering agents. Furthermore, the longer follow-up in the present study means greater potential for the recording of change over time, irrespective of cause. Certainly the variation in cholesterol over time among patients with SLE far exceeds that reported for the general population, in whom, in the absence of treatment, cholesterol levels tend to be relatively stable over time [18,19].

Likewise, almost half (46.4%) of all patients in this study had varying hypertension over the duration of the study, whereas only 1.7% had persistent hypertension. Although no previous studies exist with which to compare the proportion of SLE patients who have persistent and variable hypertension, the findings of this study support our original hypothesis that BP likely takes a variable course in patients with SLE.

The absolute total variance in TC and BP is reported in Table 4. The magnitude of total variance for TC is much smaller than that for SBP and DBP, reflecting the smaller range of possible values for the former. In addition, TC measurements may be inherently less variable over time for physiological reasons and also because TC is measured in a laboratory by using standardized assays that have small interassay variation [20]. Conversely, blood pressure measurements are subject to measurement error by physicians and volatility because of the phenomenon of 'white-coat hypertension.' Sequential studies in the general population have shown that BP can decrease by an average of 10 to 15 mm Hg between clinic visits [9,10]. Thus, many patients considered to be hypertensive at initial visits to a clinic turn out to be normotensive. To date, no studies have directly compared blood-pressure variability over time in SLE patients with healthy population controls.

Previous studies evaluated the role of TC and BP as predictors of atherosclerotic coronary events in SLE; this is the first study to look at these risk factors as 'outcome' variables and to seek to determine their independent correlates. The importance of this approach is twofold. First, this type of analysis provides insight into the reasons for the pronounced variability over time of these cardiac risk factors in SLE. Second, identifying correlates of TC and BP in SLE aids in the selection of covariates and interaction terms for inclusion in multivariate models when the outcome of interest is atherosclerotic coronary events.

In our analyses, we used GEE to allow adjustment for the expected correlation between repeated measures over time within individuals ('fixed effects'). These models have shown significant associations between increasing age and each of TC, SBP, and DBP. The association between older age and elevation in lipid levels and blood pressure is well described in the general population [21,22]. Our models have also shown that greater disease activity at the time of measurement is independently associated with higher TC, SBP, and DBP. This is a very important observation. Borba *et al. *[23] previously noted a significant correlation between SLEDAI scores and all lipid subfractions, including TC, as well as an 'active lupus pattern' of dyslipidemia in times of disease activity.

Although we found that use of immunosuppressives was significantly and independently associated with elevated TC, it is unlikely that hypercholesterolemia is a direct effect of treatment with these agents. Rather, immunosuppressive use is likely a surrogate for persistent low-grade disease activity that may not be adequately captured by the SLEDAI-2K scoring system. Notably, coincident use of immunosuppressives was negatively associated with both SBP and DBP, indicating that although greater disease activity is associated with higher BP, control of disease activity is associated with a reduction in BP.

The findings of this study support the long-suspected independent association between hypercholesterolemia and hypertension in SLE [24]. In this study, hypertension and treatment with antihypertensives were significantly associated with TC, whereas hypercholesterolemia and lipid-lowering therapy were significantly correlated with both SBP and DBP. This association highlights the phenomenon of 'clustering' of traditional cardiac risk factors within individuals with SLE and stresses the need for screening for additional cardiac risk factors when one or more risk factors are present.

As shown in previous studies, concomitant use of antimalarials was associated with lower levels of TC. Reduction in plasma cholesterol level is one of the direct pharmacologic effects of antimalarials in patients with SLE [25-27]. In this study, antimalarial use was also associated with lower levels of both SBP and DBP. However, a reduction in BP is not known to be a direct pharmacologic effect of this class of drugs. More likely, this association again points to the link between hypercholesterolemia and hypertension in SLE. Further support for this link was manifest in the association between lipid-lowering therapy and both reduced TC and BP. This observation also suggests that lipid-lowering therapy may have beneficial effects in patients with SLE, independent of a reduction in cholesterol level. However, the role of lipid-lowering therapy in prevention of atherosclerotic events in SLE can be definitively assessed only in an intervention study.

Among women with SLE, other independent correlates of TC and BP were current smoking and hormone-replacement therapy. However, our analyses were limited by lack of data on pack-years of smoking [28]. The association between smoking and hypercholesterolemia has been well described in the general population, and now, in this study, it also has been demonstrated in women with SLE [29]. In the general population, smoking also is associated with hypertension, in particular, with elevated SBP, an association that also was found in this study of patients with SLE [30]. Although among postmenopausal women, estrogen has been shown to have a beneficial effect on serum lipid concentrations, progestin contained in most standard HRT regimens partly negates this effect [28,31,32]. The net result of these opposing effects is dependent on the patient's age and overall cardiovascular risk profile. The association between diabetes and BP seen here in women with SLE has been well described in the general population [33].

The link between longer disease duration and higher TC and DBP suggests that the accrual of cardiac risk factors occurs over the course of disease and is consistent with the concept that chronic inflammation contributes to cardiac risk through association with traditional risk factors and other as-yet-undefined mechanisms.

Finally, this study has confirmed the well-known association between corticosteroid use and hypercholesterolemia [34,35]. This highlights the need for vigilant monitoring of lipid levels in times of active disease and during treatment with corticosteroids.

Future studies must be done to quantify the CAD risk associated with corticosteroid dose. Future studies will also need to determine the relation between various lipids and lipoproteins, such as high- and low-density lipoprotein cholesterol (HDL-C and LDL-C) over time in SLE. Lack of a large number of serial measurements of these lipid and lipoprotein fractions among our patients precluded us from doing such an analysis in the present study.

The contributions of this study to the field of SLE-related CAD are both conceptual and practical. First, this study has illustrated a very important concept: the marked variability of TC and BP over time in patients with SLE. The dynamic nature of these variables, in patients with SLE, makes a strong case for deriving summary measures that better capture cumulative exposure to these risk factors over time, than a single-point-in-time or 'snap-shot' measurement. Use of such cumulative measures would allow more-accurate quantification of risk for CAD in SLE. Second, this study has provided some insights into the complex relation between various risk factors for CAD in SLE. However, these interactions merit further investigation in longitudinal studies.

## Conclusions

This study has shown that TC, SBP, and DBP take a dynamic course in SLE, with more than half of the total variance over time seen within individual patients. Here we have shown that these risk factors fluctuate because of changes in disease activity, medications, and the accrual of other cardiovascular risk factors. The variable nature of cholesterol and blood pressure in patients with SLE makes a compelling case for deriving summary measures that better capture cumulative exposure to these risk factors over time.

## Abbreviations

ACR: American College of Rheumatology; ANOVA: analysis of variance; BP: blood pressure; CAD: coronary artery disease; CI: confidence interval; DBP: diastolic blood pressure; GEE: generalized estimating equation; HDL-C: high-density lipoprotein cholesterol; HRT: hormone-replacement therapy; LDL-C: low-density lipoprotein cholesterol; Max: maximum; Min: minimum; mm Hg: millimeters of mercury; mmol/L: millimoles per liter; SD: standard deviation; SLE: systemic lupus erythematosus; SLEDAI-2K: Systemic Lupus Erythematosus Disease Activity Index 2000; TC: total cholesterol.

## Competing interests

The authors declare that they have no competing interests.

## Authors' contributions

MN participated in the study design, collection and analysis of data, interpretation of results, and preparation of manuscript; DDG, in the study design, collection of data, interpretation of results, and preparation of manuscript; DI, in the study design, analysis of data, interpretation of results, and preparation of manuscript; PJH, in the study design, interpretation of results, and preparation of manuscript; and MBU, in the study design, collection of data, interpretation of results, and preparation of manuscript.
